# The microbiological characteristics and risk factors for PICC-related bloodstream infections in intensive care unit

**DOI:** 10.1038/s41598-017-10037-2

**Published:** 2017-11-08

**Authors:** Shumin Zhang, Xiaofeng Sun, Yan Lei

**Affiliations:** 1 Department of Intensive Care Unit, 306th Hospital of Chinese PLA, Beijing, 100101 China; 2 Department of Cardiovascular Surgery, 306th Hospital of Chinese PLA, Beijing, 100101 China; 3 Division of Hematology and Oncology, 306th Hospital of Chinese PLA, Beijing, 100101 China

## Abstract

The study was aimed to investigate the pathogens distribution and risk factors for PICC-related bloodstream infection in intensive care unit (ICU) patients. 402 patients placed with PICC in ICU were recruited in the study. The microbiological characteristics of PICC-related infection were investigated by Vitek 2 Compact automated microbial system. Antibiotics sensitivity was performed with disk diffusion and minimum inhibitory concentration (MIC) methods. Multivariate logistic and cox analyses were performed to identify the risk factors for PICC-related infection in ICU patients. 38 PICC-related infection cases were observed, and its morbidity was 9.45%. The morbidity was significantly higher in power PICC cases than that in common PICC cases. Gram-positive bacteria might be responsible for the major infection cases, followed by gram-negative bacteria, and fungi. Drug sensitivity analyses indicated that gram-negative bacteria showed low resistance to carbapenems antibiotics, and Cefperazone/sulbactam. The gram-positive bacterial exhibited sensitive to Teicoplanin and Vancomycin. The isolated fungi showed low resistance to the commonly used antifungal antibiotics. Multivariate analyses demonstrated that power PICC, high Charison scores, diabetes mellitus, double lumens triple lumens were risk factors for PICC-related infections among ICU patients. Power PICC, high Charison scores, diabetes mellitus, multi-lumens are risk factors for PICC-related bloodstream infection in ICU patients.

## Introduction

Central venous catheters (CVCs) are widely used in clinical setting due to their functional roles in intravenous treatments, laboratory testing, and hemodynamic monitoring^1^. Peripherally inserted central catheter (PICC), a specialized CVC, play an important role in treatment of hospitalized patients, especially intensive care unit (ICU) patients^2^. Compared with the traditional CVCs technique, PICC exerts various advantages, such as convenient placement without pleura-pulmonary damage, low cost, and long-term and stable vein access^3,4^. At present time, PICC has become widespread in clinical management for extended courses of chemotherapy, medication administration, and parenteral nutrition^5^, especially with the introduce of Power-injected PICC. Power PICC is made of ultra-resistant polyurethane which allows high flow injection using high pressure pump. Power PICC can meet the requirements for high flow rate of intravenous infusion, and multiple-lumen catheters which are common need among patients in ICU ward^6^. Thus, PICC is increasingly used in ICU setting.

Despite the various advantages of PICC for ICU patients, PICC shows some complications, including catheter-related thrombophlebitis, and bloodstream infection^7^. PICC-related bloodstream infection represents a severe complication that may lower the life of quality, increase cost, even contribute to mortality^8^. It is general considered that PICC shows low risk of bloodstream infection, compared with traditional CVCs^9–11^. However, PICC-related bloodstream infection is still a great challenge for ICU patients. To improve the management of PICC-related bloodstream infection, various researches were designed to identify the risk factors for infection among patients placed with PICC. For instance, Baxi *et al*. reported that multi-lumens and power PICC could increase the risk of PICC-related bloodstream infection^12^. A study based on cancer patients demonstrated that fixing method, indwelling season, catheter care, and tip position were significantly associated with incidence of PICC-related bloodstream infection^13^. Nevertheless, there was no consistent conclusion.

In this study, we aimed to investigate the basic characteristics of PICC-related bloodstream infections in ICU patients. Furthermore, multivariate analyses were applied to identify the risk factors for infections based on the study populations. In addition, antibiotics play pivotal roles in treatment of infection. In order to guide the empirical antibiotic application, the microbiological characteristics of PICC-related bloodstream infection was investigated, as well the drug sensitivity analysis.

## Materials and Methods

### Study population

The following methods were carried out in accordance with the approved guidelines. All authors reviewed the results and approved the final version of the manuscript. This study was approved by the Ethics Committee of the 306th Hospital of Chinese PLA.

All experimental protocols were approved by 306th Hospital of Chinese PLA, written informed consent was obtaining from every patient.

The patients in ICU who were placed PICC at 306th Hospital of Chinese PLA were collected in the study. The current investigation was based on adult group, and the expected survival time of the patients was longer than 14 days. In addition, the patients who were diagnosed with PICC placement contraindication, such as catheter allergy, suspicion of systemic infection, and so on, should be excluded from the research. Demographics information such as gender and age, and basic characteristics including Charlson scores, comorbidities, and antibiotic history were collected. In addition, the PICC characteristics such as indication for insertion, number of lumens, vein and arm of insertion, types of PICC were abstracted from the medical records. The related information of the patients were summarized in Table 1.

**Table 1:** Basic characteristics of the study population.

Parameters	Total (n = 402)	Patients with infection (n = 38,9.45%)	Patients without infection (n = 364, 90.55%)	*P* value
**Demographic**s				
Gender				0.455
Male	199 (49.50)	21 (55.26)	178 (48.90)	
Female	203 (50.50)	17 (44.74)	186 (51.10)	
Age (year)	55.99 ± 11.27	56.84 ± 10.47	55.91 ± 11.36	0.627
**Comorbidities**				
Charlson scores	6.42 ± 2.85	7.18 ± 2.72	6.34 ± 2.85	0.082
Hypertension				0.941
Yes	167 (41.54)	16 (42.10)	151 (41.48)	
No	235 (58.46)	22 (57.90)	213 (58.52)	
Diabetes mellitus				0.018
Yes	171 (42.54)	23 (60.53)	148 (40.66)	
No	231 (57.46)	15 (39.47)	216 (59.34)	
Active malignancy				0.362
Yes	173 (43.03)	19 (50.00)	154 (42.31)	
No	229 (56.97)	19 (50.00)	210 (57.69)	
Antibiotics application				0.675
Yes	214 (53.23)	19 (50.00)	195 (53.57)	
No	188 (46.77)	19 (50.00)	169 (46.43)	
**PICC characteristics**				
Duration of PICC insertion (days)	31.82 ± 23.17	25.84 ± 13.87	32.44 ± 23.86	0.095
PICC adjustments				0.200
Yes	177 (44.03)	13 (34.21)	164 (45.05)	
No	225 (55.97)	25 (65.79)	200 (54.95)	
Power PICC				0.003
Yes	215 (53.48)	29 (76.32)	186 (51.10)	
No	187 (46.52)	9 (23.68)	178 (48.90)	
Arm of PICC insertion				0.510
Left	179 (44.53)	15 (39.47)	164 (45.05)	
Right	223 (55.47)	23 (60.53)	200 (54.95)	
Vein of PICC insertion				0.294
Internal jugular	18(4.48)	4 (10.53)	14 (3.85)	
Basilic	273 (67.91)	25 (65.79)	248 (68.13)	
Brachial	84 (20.90)	7 (18.42)	77 (21.15)	
Cephalic	27 (6.72)	2 (5.26)	25 (6.87)	
Number of PICC lumen				0.001
1	163 (40.55)	7 (18.42)	156 (42.86)	
2	189 (47.01)	20 (52.63)	169 (46.43)	
3	50 (12.44)	11 (28.95)	39 (10.71)	

### PICC setting

The operating procedure for PICC placement was according to the previous description^14^. All PICC insertion was done by the same vascular access team using sterile barriers and 2% chlorhexidine in alcohol site antisepsis. PICC placement was confirmed based on the portable ultrasonography and interventional radiology, and adjusted according to the finding of the chest radiograph or fluoroscopy. Routine surveillance was performed for all the patients by the same vascular access team. PICC was flushed with 10 mL normal saline daily according to the standard protocol. When PICC was removed or the patients presented a PICC-related bloodstream infection, the follow up investigation was finished. The days for PICC use was defined as the duration between the PICC insertion and removal. The time to infection was considered as the time from PICC insertion to the date of first positive culture isolated.

### Definition of PICC-related bloodstream infections

PICC-related bloodstream infections were diagnosed based on the hospital diagnosis, nursing records, and microbiology data. The combined results were defined according to the National Healthcare Safety Network surveillance definition^15^. The blood specimens should be collected from the patients, when the patients presented with the infectious signs (fever > 38 °C, chills, rigor, hypotension) and without no other infectious sources. PICC-related bloodstream infection was defined as presence of bacteria or fungus in 1 or more blood cultures. The isolated organisms was identified by Vitek 2 Compact automated microbial system (BioMerieux Inc. ver04.02, France). The pathogen responsible for PICC-related bloodstream infection was defined as the organisms isolated from the positive blood culture. When more than one pathogens were isolated from the blood culture, the infection was considered as polymicrobial.

### Drug sensitivity test

Antibiotics sensitivity analyses were performed with a disk diffusion method according to the standards of Clinical and Laboratory Standards Institute (CLSI) and the minimum inhibitory concentration (MIC) determined by BIOMIC system (Giles Scirntific, USA).

### Statistical analysis

The rates of infection was calculated by percentage (%), moreover, we compared the percentages between Power PICC and common PICC. Demographic parameters and risk factors for PICC related bloodstream infection were summarized by descriptive statistics, and analyzed by chi-square test. Student’s t test was applied for categorical variables analysis. Logistic regression analysis was performed to identify the risk factors for PICC-related infection, and the results were expressed as odds ratios (ORs) and 95% CIs. In addition, Cox proportional hazards regression was also used to evaluate the potential risk factors for PICC-related bloodstream infection according to the time to infection. The statistical analysis were performed in SPSS 18.0 software (SPSS, Inc., Chicago, IL, USA). *P* value less than 0.05 was considered statistically significant.

## Results

### Basic characteristics of the study population

402 ICU patients including 199 men and 203 women were enrolled in the investigation. The average age of the patients were 55.99 ± 11.27 years. Among the patients, 167 (41.54%) of them were diagnosed with hypertension, 171 (42.54%) patients were combined with diabetes mellitus, and malignancy cases accounted for 43.03%. Furthermore, 214 (52.23%) cases were with antibiotics application history (Table 1).

The average PICC insertion time for the patients was 31.82 ± 23.17 days, and 215 (53.48) patients received power PICC. The major patients were placed single lumen (40.55%, n = 163) and double lumens (47.01%, n = 189), while only 12.44% (n = 50) patients were placed triple lumens. PICC was most commonly inserted in right arm (55.47%, n = 223) and basilic vein (67.91%, n = 273) (Table 1).

### Occurrence rate of PICC-related bloodstream infections

Among the eligible patients, 38 patients (9.45%) presented PICC-related bloodstream infection. Moreover, the morbidity of infection was significantly higher in patients placed power PICC than those receiving common PICC (13.49% vs 4.81%, *P* = 0.003).

### Microbiological characteristics of PICC-related infections

The distributions of pathogens were analyzed for the patients. Analysis results demonstrated that gram-positive bacteria was responsible for the major infection cases (60.53%, n = 23), followed by gram-negative bacteria (31.58%, n = 12). There were 3 (7.89%) infection episodes caused by fungi. The isolated gram-positive bacteria mainly included *Staphylococcus aureus* (n = 10), *Enterococcus spp* (n = 5), *Staphylococcus haemolyticus* (n = 3), *Staphylococcus epidermidis* (n = 3), and *Staphylococcus huminis* (n = 2). Furthermore, 2 methicillin-resistant *Coagulase negative staphylococci* and 5 methicillin-resistant *Staphylococcus aureus* were identified. Among the gram-negative bacteria, *Escherichia coli* accounted for 5 cases, *Klebsiella pneumoniae* accounted for 3 cases, and there were 2 *Pseudomonas aeruginosa* cases and 2 *Acinetobacter baumanii* episodes. The isolated fungi included *Candida glabrata* (n = 1), *Candida albicans* (n = 1), and *Candida parapsilosis* (n = 1) (Table 2).

**Table 2:** The microbiological distributions of PICC-related infections.

Pathogens	Case number	Percentages (%)
**Gram-negative bacteria**	12	31.58
*Escherichia coli*	5	13.16
*Klebsiella pneumoniae*	3	7.89
*Pseudomonas aeruginosa*	2	5.26
*Acinetobacter baumanii*	2	5.26
**Gram-positive bacteria**	23	60.53
*Enterococcus spp*	5	13.16
*Staphylococcus epidermidis*	3	7.89
*Staphylococcus huminis*	2	5.26
*Staphylococcus haemolyticus*	3	7.89
*Staphylococcus aureus*	10	26.32
Methicillin-resistant *Coagulase negative staphylococci*	2	5.26
**Fungi**	3	7.89
*Candida glabrata*	1	2.63
*Candida albicans*	1	2.63
*Candida parapsilosis*	1	2.63
Total	38	100

### Antibiotics sensitivity analysis for the isolated pathogens

We also investigated the sensitivity of the isolated pathogens to the commonly used antibiotics. The results indicated that gram-negative bacteria were sensitive to carbapenems antibiotics (resistant rate 8.33%), amikacin (8.33%), Cefperazone/sulbactam (25.00%) and Piperacillin/tazobactam (33.33%). The gram-positive bacterial exhibited sensitive to Teicoplanin (0.00%) and Vancomycin (4.35%). Additionally, the isolated fungi showed low resistance to the commonly used antifungal antibiotics (Table 3).

**Table 3:** The antibiotics sensitivity analysis for isolated pathogens.

**Antibiotics**	**Number of resistance cases (n, %)**				
	***Escherichia coli*** **(n = 5)**	***Klebsiella pneumoniae*** **(n = 3)**	***Pseudomonas aeruginosa*** **(n = 2)**	***Acinetobacter baumanii*** **(n = 2)**	**Total (%)**
**Gram-negative bacteria**					
Cefepime	3 (60.00)	2 (66.67)	1 (50.00)	2 (100.00)	66.67
Ceftazidime	4 (80.00)	3 (100.00)	1 (50.00)	2 (100.00)	83.33
Ceftriaxone	5 (100.00)	2 (66.67)	2 (100.00)	2 (100.00)	91.67
Imipenem	1 (20.00)	0 (0.00)	0 (0.00)	0 (0.00)	8.33
Meropenem	0 (0.00)	1 (33.33)	0 (0.00)	0 (0.00)	8.33
Levofloxacin	2 (40.00)	1 (33.33)	1 (50.00)	0 (0.00)	33.33
Cefperazone/sulbactam	2 (40.00)	0 (0.00)	1 (50.00)	0 (0.00)	25.00
Piperacillin/tazobactam	3 (60.00)	1 (33.33)	0 (0.00)	0 (0.00)	33.33
Amikacin	0 (0.00)	1 (33.33)	0 (0.00)	0 (0.00)	8.33
	***Coagulase negative staphylococci*** **(n = 8)**	***Streptococcus species*** **(n = 10)**	***Enterococcus spp*** **(n = 5)**		
**Gram-positive bacteria**					
Penicillin	8 (100.00)	10 (100.00)	5 (100.00)	100.00	
Erythromycin	8 (100.00)	8 (80.00)	4 (80.00)	86.96	
Gentamicin	6 (75.00)	9 (90.00)	4 (80.00)	82.61	
Levofloxacin	5 (62.50)	3 (30.00)	2 (40.00)	43.48	
Vancomycin	0 (0.00)	1 (10.00)	0 (0.00)	4.35	
Teicoplanin	0 (0.00)	0 (0.00)	0 (0.00)	0.00	
	***Candida glabrata*** **(n = 1)**	***Candida albicans*** **(n = 1)**	***Candida parapsilosis*** **(n = 1)**		
**Fungi**					
Fluconazole	0 (0.00)	0 (0.00)	0 (0.00)	0.00	
Amphotericin B	1 (100)	0 (0.00)	0 (0.00)	33.33	
Itraconazole	0 (0.00)	0 (0.00)	0 (0.00)	0.00	
Voriconazole	1 (100)	0 (0.00)	0 (0.00)	33.33	
Fluorocytosine	1 (100)	0 (0.00)	0 (0.00)	33.33	

### Risk factors for PICC-related bloodstream infections in ICU patients

We compared the medical parameters between infection cases and non-infection patients. As shown in Table 1, diabetes mellitus (*P* = 0.018), PICC type (*P* = 0.003), and number of PICC lumens (*P* = 0.001) were significantly different between the study groups.

**Figure 1:**
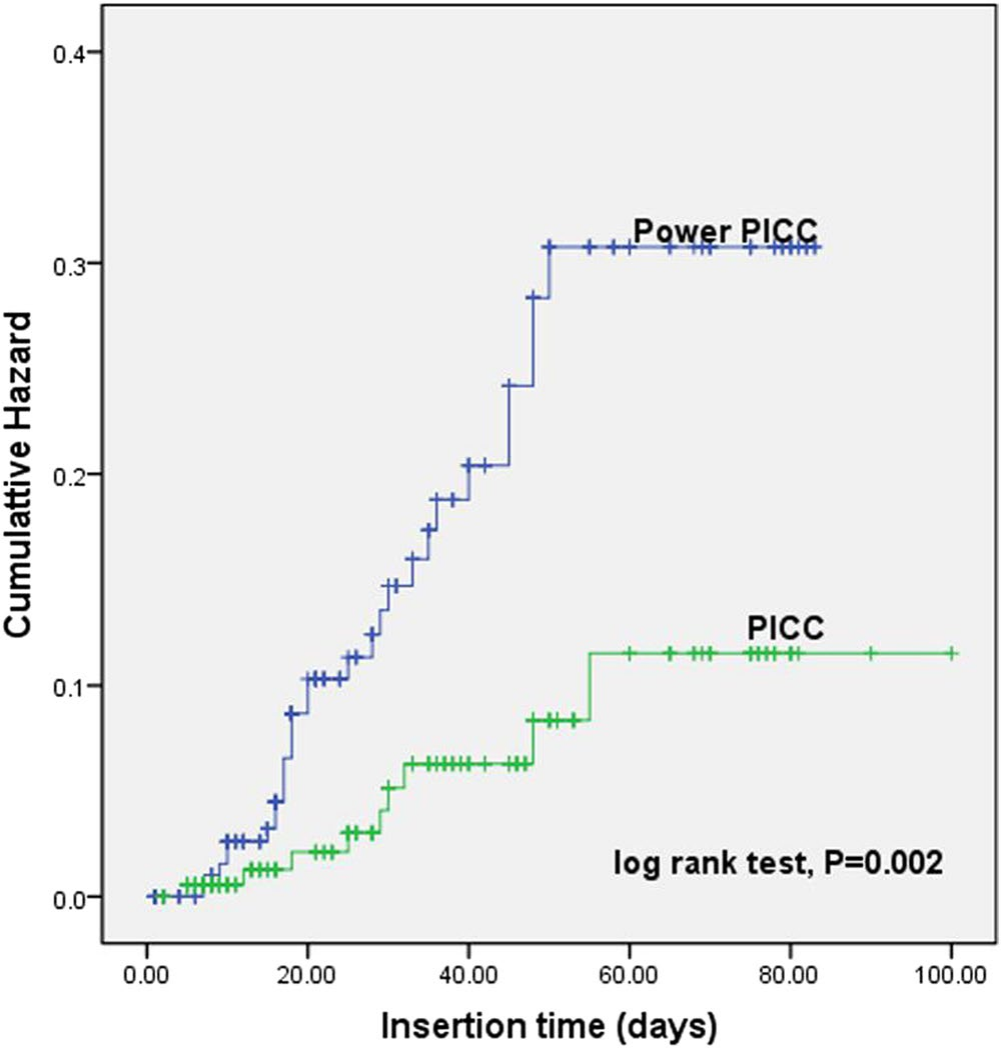
The cumulative hazard curves for PICC-related bloodstream infection were plotted based on the types of PICC. The curves suggested that patients placed with power PICC had a significantly higher occurrence of infection than those receiving common PICC (log rank test, *P* = 0.002).

**Figure 2:**
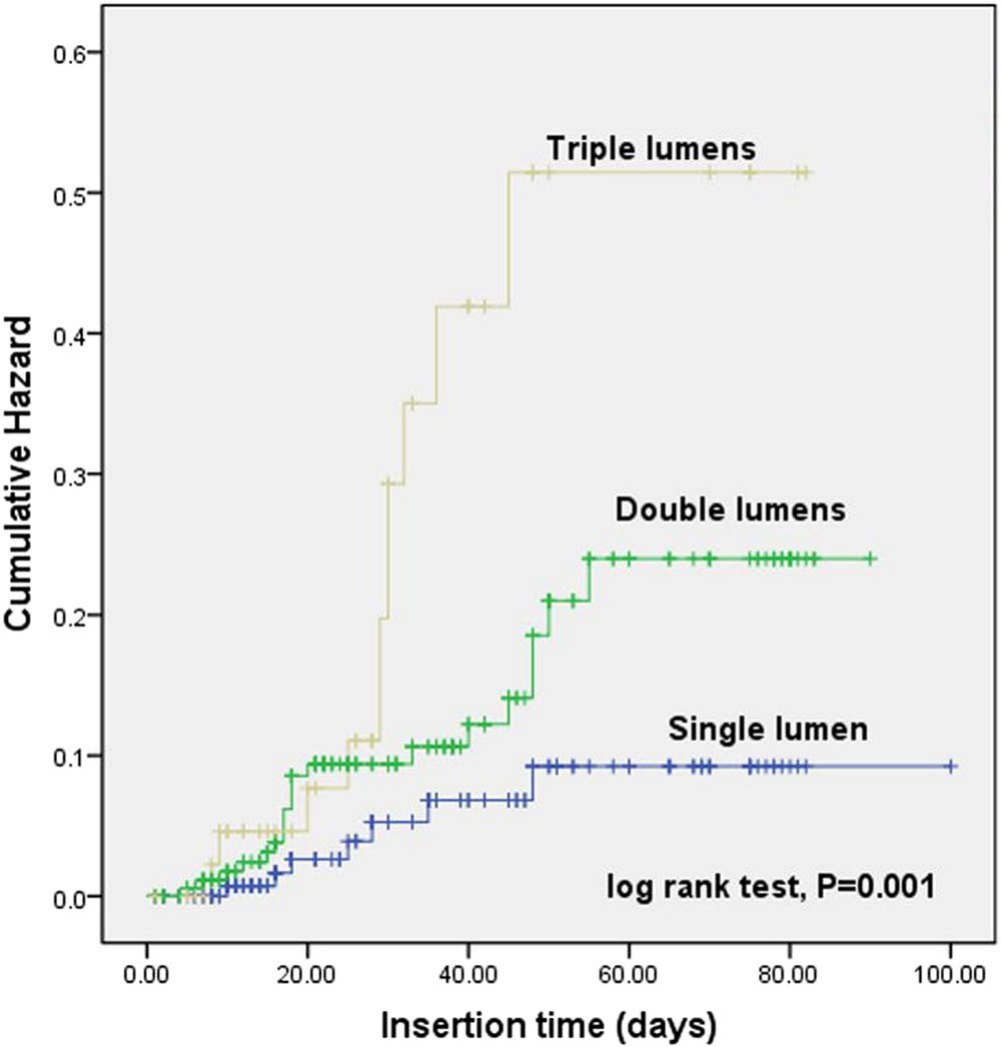
The cumulative hazard curves for PICC-related infection were constructed according to the number of lumens. Analysis results indicated that compared with double and triple lumens, single lumen would lower the risk of infection in ICU patients (log rank test, *P* = 0.001).

In addition, the cumulative hazard curves of PICC-related bloodstream infection was constructed based on the types of PICC and number of lumens. As shown in Fig. 1, the morbidity of infection was significantly higher in patients placed power PICC than those receiving common PICC (log rank test, *P* = 0.002). The curves constructed according to the number of lumens demonstrated that single lumen would lower the risk of PICC-related infection in ICU patients (log rank test, *P* = 0.001) (Fig. 2).

In order to identify the risk factors for PICC-related bloodstream infections, multivariate logistic and cox analyses were performed in the current study. Logistic analyses demonstrated that power PICC (OR = 4.239, 95%CI = 1.857–9.678, *P* = 0.001), high Charison scores (OR = 1.137, 95%CI = 1.004–1.287, *P* = 0.044), diabetes mellitus (OR = 2.663, 95%CI = 1.293–5.482, *P* = 0.008), double lumens (OR = 3.352, 95%CI = 1.343–8.368, *P* = 0.010) and triple lumens (OR = 8.018, 95%CI = 2.771–23.202, *P* = 0.000) were risk factors for infection among ICU patients placed PICC. The results of multivariate cox analysis suggested that power PICC (HR = 4.197, 95%CI = 1.932–9.119, *P* = 0.000), high Charison scores (HR = 1.120, 95%CI = 1.001–1.253, *P* = 0.048), diabetes mellitus (HR = 2.370, 95%CI = 1.224–4.588, *P* = 0.011), double lumens (HR = 2.939, 95%CI = 1.233–7.010, *P* = 0.015) and triple lumens (OR = 6.352, 95%CI = 2.533–16.580, *P* = 0.000) were independent risk factors for PICC-related infections (Table 4).

**Table 4:** Multivariate logistic and Cox analysis for risk factors of PICC-related infections in ICU patients.

Parameters	Logistic analysis		Cox analysis	
	OR (95%CI)	*P*	OR (95%CI)	*P*
Power PICC	4.239 (1.857–9.678)	0.001	4.197 (1.932–9.119)	0.000
Charison scores	1.137 (1.004–1.287)	0.044	1.120 (1.001–1.253)	0.048
Diabetes mellitus	2.663 (1.293–5.482)	0.008	2.370 (1.224–4.588)	0.011
Lumens				
1	Reference		Reference	
2	3.352 (1.343–8.368)	0.010	2.939 (1.233–7.010)	0.015
3	8.018 (2.771–23.202)	0.000	6.352 (2.433–16.580)	0.000

## Discussion

At present time, the application of PICC is prevalent in ICU setting. PICC can provide long-term, stable vascular access for medications, parenteral nutrition, and hemodynamic monitoring^16^. Moreover, PICC can be placed by the specially trained nurses in outpatient department, causing low cost compared with the traditional CVCs^17^. However, the wide application of PICC may lead to several complications, such as catheter-related thrombophlebitis, and bloodstream infection^18^. PICC-related infection represents a severe complication that may contribute to prolonged hospital stay, increased medical cost, even mortality^8,19^. In this study, we investigated the basic and microbiological characteristics of PICC-related bloodstream infection, in order to prevent the incidence of infection and improve the management of PICC-related bloodstream infection.

In the present study, 402 eligible ICU patients were recruited, and 38 of them developed to PICC-related infection. The morbidity of PICC-related infection was 9.45%. Furthermore, we found that the occurrence rate of infection was significantly higher in patients receiving power PICC. Power PICC provide high flow rate of infusion, easy central venous pressure monitoring, and high-pressure injection of contrast media for radiodiagnostic procedures, and multi-lumens that is widely used in ICU as common practice^20^. Unfortunately, we found that the application of power PICC could increase the risk of PICC-related bloodstream infection, moreover, multivariate analyses proved that it was a risk factor for infection in ICU patients. Baxi *et al*. reported that power PICC was correlated with increased risk of PICC-related bloodstream infection^12^. Chopra *et al*. confirmed that power PICC was a risk factor for PICC-related bloodstream infection^21^. The two investigations were consistent with our study. However, some researches held opposing viewpoints. The study carried out by Pittiruti *et al*. demonstrated that the maintenance of power PICC exerted significant advantages in lowering the occurrence of infectious and non-infectious complications^22^. Thus, further researches were still needed to address the issue.

Compared with common PICC setting, power PICC can provide multi-lumens for simultaneous administration of potentially incompatible drugs^6,22^. However, growing evidences have indicated that multi-lumens was associated with increased risk of PICC-related complications. It was reported that multi-lumens application was correlated with unacceptable high venous thrombosis rates^23,24^. The influence of multi-lumens on incidence of PICC-related bloodstream infection was also reported in the previous studies. The related studies demonstrated that multi-lumens could increase the morbidity of infection among patients placed with PICC^12,21^. In this study, multivariate analyses demonstrated that double and triple lumens were significantly associated with extremely high occurrence of PICC-related bloodstream infection in ICU patients. Taken together, multi-lumens was a risk factor for PICC-complications, and serious monitoring should be taken when multi-lumens PICC was performed for ICU patients. In addition, there was an interesting finding in our research that diabetes mellitus was still a risk factor for PICC-related infection among ICU patients. The association between diabetes mellitus and PICC-related infection had been rarely reported in the previous investigation. The issue was needed to be addressed by further researches.

Timely and appropriate empirical antibiotic treatment is pivotal for outcomes of patients with bloodstream infection^25,26^. In order to improve the therapeutic effects of antibiotics, it is necessary to investigate the epidemiology of PICC-related bloodstream infection. In this study, we found that gram-positive bacterial such as *Staphylococcus aureus*, and *Enterococcus spp*, might be responsible for the major infection cases, followed by gram-negative bacteria including *Escherichia coli* and *Klebsiella pneumoniae*, and fungi. Drug sensitivity analyses demonstrated that gram-negative bacteria were sensitive to carbapenems antibiotics, amikacin, Cefperazone/sulbactam and Piperacillin/tazobactam. The gram-positive bacterial exhibited similarly high sensitive to Teicoplanin and Vancomycin. Additionally, the isolated fungi showed low resistance to the commonly used antifungal antibiotics. The antibiotics shown above might be used for empirical treatments for PICC-related infection among ICU patients in our hospital.

In conclusion, PICC-related bloodstream infection is a great challenge for ICU patients. Gram-positive and gram-negative bacteria may represent the major reasons for PICC-related infection. Power PICC, diabetes mellitus, high Charison scores, and multi-lumens may increase the risk of PICC-related infections among ICU patients.
